# From 2D kaolinite to 3D amorphous cement

**DOI:** 10.1038/s41598-024-81882-1

**Published:** 2025-01-11

**Authors:** Juan A. G. Carrio, Ricardo K. Donato, Alexandra Carvalho, Gavin K. W. Koon, Katarzyna Z. Donato, Xin Hui Yau, Dmytro Kosiachevskyi, Karen Lim, Vedarethinam Ravi, Josny Joy, Kelda Goh, Jose Vitorio Emiliano, Jerome E. Lombardi, A. H. Castro Neto

**Affiliations:** 1 Centre for Advanced 2D Materials, National University of Singapore, Singapore, 117546 Singapore; 2 Institute for Functional Intelligent Materials (I-FIM), National University of Singapore, Singapore, 117544 Singapore; 3 Department of Materials Science and Engineering, National University of Singapore, Singapore, 117575 Singapore; 4 Present Address: Institute of Inorganic Chemistry of the Czech Academy of Sciences, Husinec-Řež 1001, 25068 Řež, Czech Republic

**Keywords:** Clays, 2D materials, Metakaolin, Alkali-activation, Geopolymer, Materials science, Nanoscale materials, Two-dimensional materials

## Abstract

Kaolinite is a single 2D layer of kaolin or metakaolin (MK), common clays that can be characterized as layered 3D materials. We show that because of its chemical composition, kaolinite can be converted into an amorphous 3D material by chemical means. This dimensional transformation is possible due to the large surface to volume ratio and chemical reactivity of kaolinite. We investigate the formation and influence of quasi- or nanocrystalline phases in MK-based alkali-activated materials (AAM) that are related to the Si/Al ratio. We analyze the formation of an AAM from a MK precursor, which is a 3D bonded network that preserves the layered structure at the nanometer scale. We also exfoliate the remaining layered phase to examine the effects of the alkali-activation in the final sheet structures embedded within the amorphous network. The final material can be used as a cement with no carbon dioxide produced by the transformation reaction.

**Supplementary Information:**

The online version contains supplementary material available at 10.1038/s41598-024-81882-1.

## Introduction

Portland cement is an invention from the early nineteenth century. The first patent from Joseph Aspdin was issued on 21 October 1824. Hence, Portland cement technology will celebrate 200 years in 2024. There is no question that this invention is one of the most important for mankind and possibly one of the oldest and prevalent technologies that are still used to this date. However, looking into a historical context, this technology is a fruit of the Industrial Revolution that started in the eighteenth century^1,2^. It was created at a time where environmental concerns were not considered important. As such, Portland cement technology is wasteful and polluting as the Industrial Revolution itself. Therefore, Portland cement manufacturing is responsible for an enormous amount of carbon dioxide emissions in the atmosphere (measured in tens of giga metric tons)^3^. In the last 30 years, research, development, and commercial activities related to alkali-activated concrete have increased due to the growing awareness of the high carbon footprint associated with Portland cement^2^. However, accounts describing the different morphological stages of green cement production, from a crystalline 2D to an amorphous 3D inorganic material, are limited. We approach kaolinite’s journey through the lenses of 2D materials, to better understand what is left of its original composition and structure in the final cementitious 3D material.

The forefront of industrial applications of 2D materials is usually represented by graphene and its derivatives. Graphene is obtained from a 3D layered material, graphite, in the same way kaolinite is obtained from a layered material, kaolin/metakaolin. Graphene^4^ and kaolinite^5^ belong to a large family of 2D materials that includes transition metal dichalcogenides, boron-nitride, among others. However, a number of challenges have hindered the adoption of graphene derivatives in commercial applications, where standardization, quality variation, and high production costs are among the most prominent^6^. Consequently, graphene-related materials are mainly used as additives because their cost is still higher than other bulk raw materials such as common ceramics, metals and polymers^7,8^. As a consequence, a search for readily available materials that can be exfoliated down to 2D is an important area of research^9–12^.

An important family of layered materials is the clays. Clays have been used for millennia in pottery and construction, and cementitious gels, which have been found in ancient Roman concrete^13^. The development of alkali-activated materials (AAMs) using clay precursors began in the nineteenth century and accelerated at the beginning of the twentieth century^14^. But the recent growth of awareness of the high carbon footprint associated with Portland cement has boosted research, development, and commercial activities related to alkali-activated concrete^2^.

Clays are layered structures made of sheet-like monolayers with one dimension in the nanometer range, which can be classified as another large family of 2D materials. The joint nomenclature committees (JNC) of the Association Internationale pour l’Etude des Argiles (AIPEA), and the Clay Minerals Society define clay minerals as either natural or synthetic, phyllosilicates or non-phyllosilicates. Traditionally classified under silicates, clay minerals have more oxygen than Si, Al, and Mg in their chemical compositions so that they can be regarded as layered hydroxides^15^. The layered structure of the phyllosilicates consists of sheets made of tetrahedra (T) and octahedra (O) that form TO or TOT layers, which have a mainly negative surface charge. In the layered double hydroxides of metal ions (LDH), the structure consists of an octahedral (O) sheet with a positive surface charge, but the layers can also be uncharged, as in talc and pyrophyllite^16^. The tetrahedra are formed by a cation coordinated to four oxygen atoms, which are sheared in three corners with adjacent tetrahedra. They form an infinite 2D hexagonal pattern along the a and b crystallographic directions^17^. In the octahedra, a cation is coordinated to six oxygen atoms and the edges are sheared to neighbor octahedra. The ratio of T/O sheets, along with their charge, results in a large variety of clays^16^.

Clays have been studied from very different perspectives of geology, mineralogy, chemistry, physics, and biology, and are still the object of intensive research. Abundant, inexpensive, readily suitable for large-scale industrial applications, and environmentally friendly (either in their natural form or after modifications)^16^, clays have found applications as sustainable functional materials in de-polluting agents, bio-compatible composites, and construction materials. Some clays, particularly those belonging to the kaolinitic branch, are aluminosilicate precursors that result in a three-dimensional (3D) gel when subjected to alkali activation, which can lead to a robust material with high mechanical strength^18^.

The sheet-like structure of kaolinite (Al_2_Si_2_O_5_(OH)_4_), the major component in kaolin, has been explored to obtain sheets with thicknesses in the nanometer range. Processes such as intercalation-exfoliation, chemical and physical exfoliation can yield kaolinite with thickness down to 1.3 nm, allowing for the fabrication of membranes made from the 2D sheets^19,20^. Calcination, acid leaching, and ultrasonic dispersion can be applied to raw kaolin to obtain modified kaolinite nano-layers^21^, which are reported in applications such as drug delivery for cancer treatment^22^. The well-known exfoliation methods used to obtain graphene and 2D materials have been derived, in part, from delamination techniques employed in the clay industry^23,24^. For example, a common technique in nanotechnology for controlling the growth and agglomeration of nanoparticles and 2D materials is using polymer-surfactant complexes of opposite charges^25^. Kaolinite can be exfoliated to individual 1:1 layers by intercalating organic solvents, urea, and polymeric compounds (such as oppositely charged polyelectrolytes)^26^, modifying its surface.

**Fig. 1:**
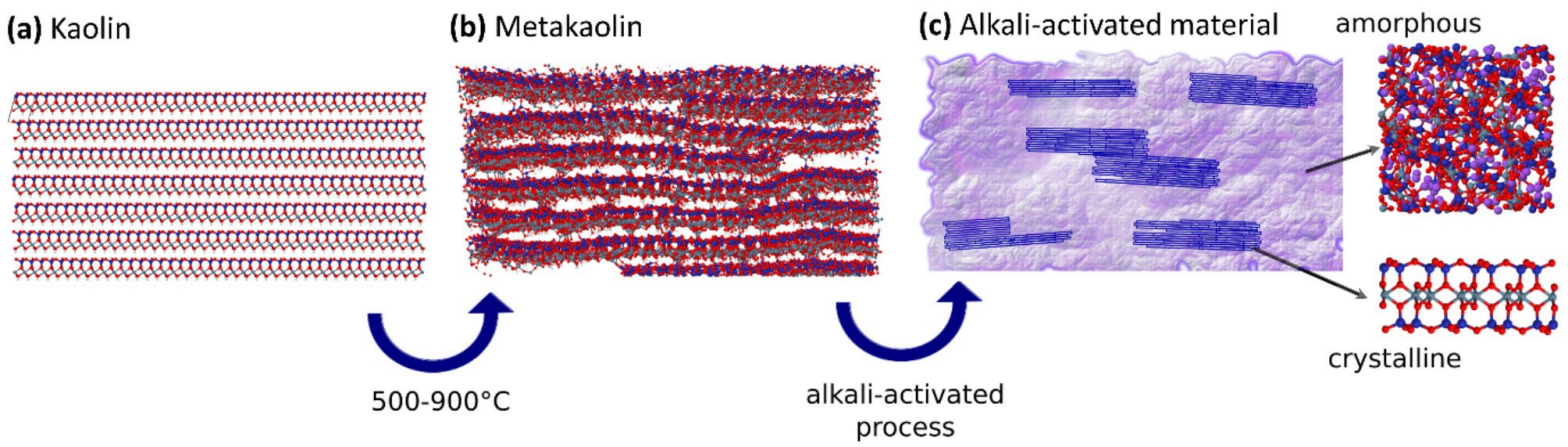
Schematic representation of the transformations involved in producing a 3D amorphous material from 2D crystalline kaolinite. (**a**) Kaolin structure followed by (**b**) its thermal conversion into metakaolin, and (**c**) alkali-activation into a composite material containing an amorphous 3D matrix and nanometer 2D crystalline domains.

We explore the relationship between the structure and bonding energetics in kaolinite, metakaolin (MK), and alkali-activated derived materials (AAM). We start by revisiting the change of structure in kaolinite/metakaolin transformation. For that, we investigate the presence of quasi- or nanocrystalline phases in MK-based AAM, which has been reported as being directly related to the Si/Al ratio due to the formation of zeolite-like phases^27–30^. Subsequently, we analyze the formation of an AAM from a MK precursor, a 3D bonded network that preserves the layered structure at the nanometer scale. Finally, we exfoliate the remaining layered phase to examine the effects of the alkali-activation in the final sheet structures, as shown in Fig. 1.

## Results and discussion

### From kaolin to metakaolin

The structure of kaolinite consists of a single sheet with AlO_6_ octahedra on one side and SiO_4_ tetrahedra on the other. The layers are stacked with an offset to form a triclinic symmetry with the space group C1^31^. The vertex-sharing SiO_4_ tetrahedra (T) form rings of six silicates, and the edge-sharing AlO_6_ octahedra (O) form rings of four aluminates. This configuration gives rise to its classification as a 1:1 T:O dioctahedral planar hydrous phyllosilicate. The term dioctahedral is due to the valence 2 of the Al^2+^ cations in the octahedra centers. Strong ionic and covalent bonds join the TO sheets of silicates and aluminates through the apical oxygens, but between TO layers, the connection is through hydrogen bonds^32^ (Fig. 1a).

Due to the hydrogen bonds, the interlayer interaction is stronger than in graphite. We define the exfoliation energy of the crystal as the energy difference between the per layer energy, *E*(bulk crystal), of the bulk material and that of a free single layer, *E*(single layer): *E*_exf_ = *E*(bulk crystal)—*E*(single layer). Thus, the exfoliation energy of kaolin is − 38 meV/Å^2^ (Table 1), compared to − 28.7 meV/Å^2^ for graphite^33^. Individual kaolinite layers are obtained at room temperature, but with a curved morphology, due to the defects in matching between the tetrahedral and octahedral sheets. For example, exfoliation of kaolinite and dickite in organic solvents has resulted in the formation of folded structures and mineral nanotubes^26^.

**Table 1:** Calculated lattice parameters and exfoliation energies (per layer) for kaolinite and MK using Density Functional Theory (DFT).

	DFT functional	*a* (Å)^1^	*b* (Å)^1^	*c* (Å)^1^	α (°)^1^	β (°)^1^	γ (°)^1^	*E*_exf_ (meV/Å)^2^
Kaolin (bulk)	PBE	5.21	9.05	7.45	92	105	90	-38
Kaolinite (ML)		5.17	8.94	-	-	-	90	
MK (bulk)		5.21	8.75	14.46	90	105	87	-31
MK (ML)		5.18	8.90	-	-	-	90	
Kaolin (bulk)	vdW-LMKLL	5.27	9.14	7.50	92	105	90	-22
Kaolinite (ML)		5.24	9.07	-	-	-	90	
MK (bulk)		5.19	8.65	14.54	89	105	87	-32
MK (ML)		5.18	8.89	-	-	-	85	
Kaolin (bulk)^35^	B3LYP-D*N							-29
Kaolin (bulk, exp.)		5.153	8.941	7.390	91.92	105.046	89.79	

^1^Cell parameters, where: a, b and c are the length of the unit cell lattice vectors, α is the angle between b and c, β is the angle between a and c, and γ the angle between a and b. The MK lattice parameters are average values for MK obtained from a 2 × 2x2 kaolin unit cell. Experimental lattice parameters are from the literature^36^ and the DFT functionals used in this study are identified in the Methods Sect. ^2^*E*_exf_ is the exfoliation energy.

Metakaolin (Al_2_O_3_.2SiO_2_) is the product of the thermal treatment of kaolinite. The complex transformation from kaolin to MK at the atomic level has been studied by least-squares real-space refinement using neutron pair distribution function (PDF) data and energy minimization with density functional modelling^34^ and molecular dynamics simulations^32^. The thermal treatment causes a de-hydroxylation process with subsequent structural transformations. The loss of hydroxyl groups on the surface of the inter-layer spacing causes the aluminum to migrate into the vacant sites of the inter-layer spaces inducing a strain in the aluminum sites, so the aluminum atoms reorganize to relieve the strain. Consequently, all atoms shift their positions, which leads to the buckling of both AlO_6_ and SiO_4_ layers. The most significant degree of reorganization corresponds to the AlO_6_ layers. The SiO_4_ layers tend to retain their configuration and accommodate according to the changes of the AlO_6_ layers. Additionally, partial transformation from O to T aluminum coordination has been observed^34^.

**Fig. 2:**
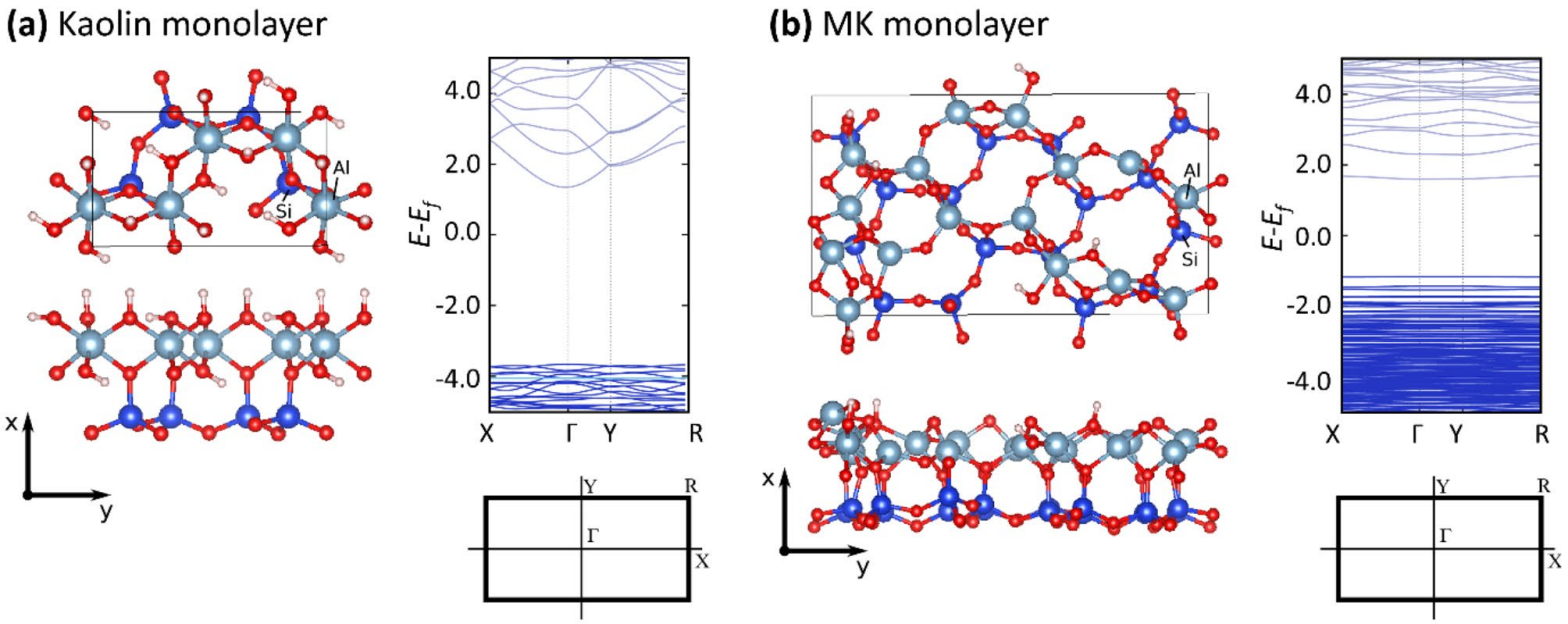
Structures and electronic band structures of (**a**) kaolin monolayer (kaolinite) and (**b**) MK monolayer obtained from DFT-PBE calculations. Insets show the rectangular Brillouin zone.

The buckling of the layers (Fig. 1b) is responsible for the atomic disorder and the subsequent absence of Bragg peaks in the X-ray diffraction pattern. Thus, MK maintains short-range order with the original layering of kaolin up to around 920°C^37^. Even so, without diffusion of the cations across the layers, the structure still retains the 2D layers inherent in kaolinite but with corrugation. The remaining micro-crystalline structure of MK has been revealed using XRD and EDX (see Sections "Alkali-activated material (AAM) from metakaolin (MK)" and "Crystalline phase composition of MK and AAM", respectively). Transmission electron diffraction patterns of the kaolin treated at 500–800 °C show 2D pseudo-hexagonal arrays of spots corresponding to the (hk0) reflections, even when the (001) reflection, corresponding to the third dimension, has disappeared^38^. Figure 2 presents the crystalline and electronic band structure of a single layer of MK, obtained from simulations (see experimental section).

The low bending rigidity of MK leads the system to form crumpled structures even at room temperature. Nevertheless, a two-dimensional order in particles with sizes up to around 100 nm is still present. Small Angle Scattering (SAXS) reveals a scattered intensity dependence, which is caused by particles with sizes of 10 nm to 100nm^39^.

**Fig. 3:**
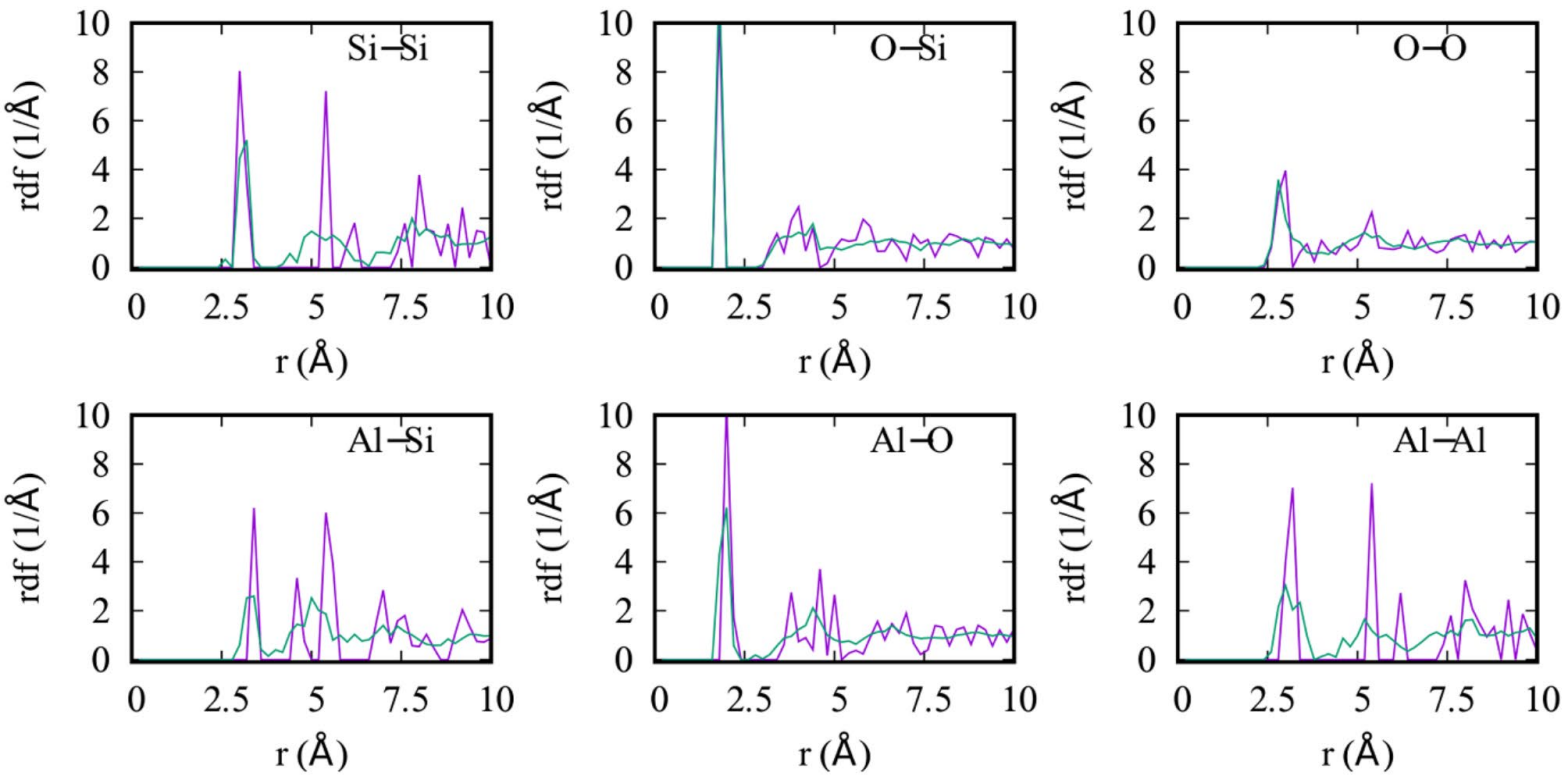
The radial distribution functions (RDF) of kaolinite (purple) and MK (green) for different atomic bonds, showing the loss of the long-range order (> 5 Å) in MK.

At the < 1 nm scale, the average lattice parameters of MK reveal that the dehydration of kaolin results in nearly no change of the layer area. The distance between layers is reduced by about 0.2 Å in average, and the exfoliation energy is slightly reduced to -31 meV/Å^2^ (Table 1), due to the loss of hydrogen bonds. The radial distribution functions (RDF), shown in Fig. 3, reveal a contrast between the crystalline kaolinite structure and the more disordered structure of MK. The aluminum layer shows more disorder than the silicon layer. While the Si–O bond peak at 1.80 Å remains undisturbed, the Al-O peak at 1.99 Å gains a shoulder at shorter lengths due to the change in Al coordination from octahedral to tetrahedral. The Si–Si and Al-Al nearest neighbor distances at 3.01 Å and 3.19 Å, respectively, are maintained due to the rigidity of the Si–O-Si and Al–O–Al units. However, the peaks corresponding to second nearest Si–Si or Al-Al neighbors are strongly reduced (at 5.40 Å and 5.39 Å) due to the disorder in the Si and Al hexagonal rings. In general, the order associated with the shorter bonds below 2.5 Å is preserved, but no order remains above second nearest-neighbor distances.

As shown in Fig. 2, both kaolinite and MK are wide bandgap insulators, with DFT-PBE bandgaps of 5.0 eV and 2.8 eV, respectively. The reduction of the electronic gap implies a reduction of the material’s work function and electron affinity and, hence, a substantial increase in its chemical reactivity. The valence band is predominantly localized on the oxygen 2p orbitals, whereas the conduction band is made up predominantly of Al and Si states. The smaller bandgap of MK and there are flat bands at the gap edges, corresponding to highly localized states. The removal of the hydrogen attached to the surface oxygen atoms leads to the formation of additional Al-O bonds, resulting in penta-coordinated aluminium, which has been argued to be partially responsible for the high reactivity of metakaolin^40^.

### Alkali-activated material (AAM) from metakaolin (MK)

The process of alkali activation of MK usually involves a series of reactions between the MK powder precursor and alkali cations in a high pH aqueous solution (NaOH, KOH, Na or K liquid silicates, or a combination thereof). After mixing MK and alkaline solution^41^, the reaction mechanism of MK-based AAM comprises of 4 main steps^27^: (*1*) MK dissolution and release of Si and Al species into solution, a process in which Al(V) and Al(VI) turn into Al(IV); (*2*) the reaction of the Si and Al species with various silicate units present in the alkali solution to form aluminosilicates oligomers; (*3*) formation of a gel-like, amorphous phase through reorganization (polymerization and gelation); (*4*) a final process of polycondensation that, depending on temperature and composition, results in a structurally stable 3D matrix.

Using the above-mentioned process, we obtained an alkali-activated material (AAM) from MK and applied it to the formation of a mortar (see experimental section). The effect of the GGBFS and slag, minor components containing Ca, is to increase the final compressive strength of the material [Cement and Concrete Composites 137 (2023) 104925].The atomic compositions of product and precursor were investigated using ICP-OES, yielding a Si:Al mass ratio of 0.38 for the MK precursor, and 5.8 for the AAM. However, this does not reflect the compositions at the crystalline phases and is a direct effect of the slag used, as will be discussed in Section "Crystalline phase composition of MK and AAM". The congruent (proportional Si/Al dissolution) or incongruent (excess Al dissolved) nature of MK dissolution is related to the alkali metal present in the activating solution. Sodium favors superficial and intralayer dissolution (congruent), whereas Potassium, which has a larger ionic size, favors a lateral-to-center dissolution favoring dealumination (incongruent)^42^. Here, we use an activator comprising both (Na/K ~ 7 mol/mol). The alkaline-earth metals found in our AAM (mostly Ca, limited Mg) come from the slag we added. The complete bulk atomic compositions can be found in Supplementary Information (SI, Table S1).

**Fig. 4:**
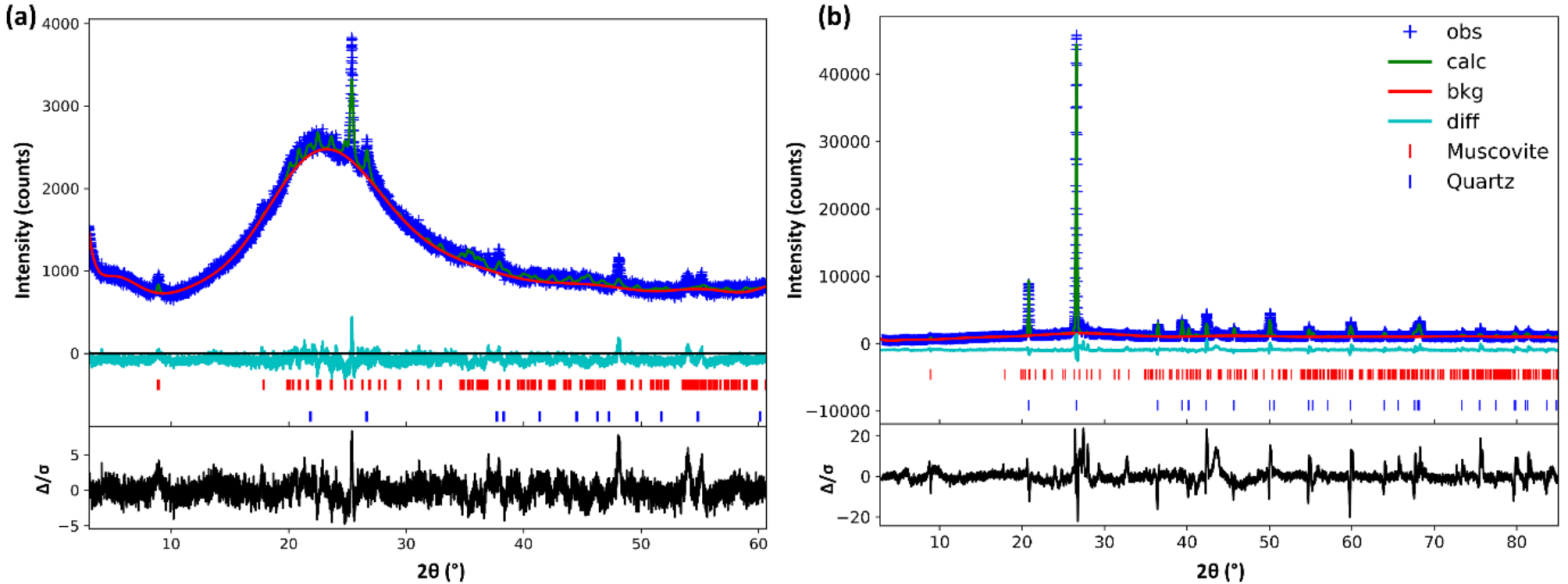
Diffractograms of MK (**a**) and AAM (**b**) with phase identification and Rietveld refinement.

X-ray diffraction (XRD) analysis was carried out considering the potential presence of kaolinite, quartz, muscovite, anatase, microcline, montmorillonite, illite, anatase and phengite phases, which have been reported in the literature^39,43^ , for the precursor MK and the resulting AAM. Both the MK and AAM contain SiO_2_ (α-quartz) and muscovite, and their diffractograms and Rietveld refinement are shown in Fig. 4a and b, respectively. The quartz can be due to the presence of sand in the mortar, or nanocrystal formation during alkali activation, as discussed later.

The quantitative phase composition and the final residuals of the Rietveld refinements extracted from Fig. 4 (details in the experimental section) are presented in Table 2. The refined structural and microstructural parameters of muscovite phase (cell parameters, atomic positions and fractions, atomic angles, atomic distances, size and strain) are reported in the Supplementary information, where we provide Crystallographic Information Files (CIF).

**Table 2:** Phase compositions and refinement residuals for the samples of MK and AAM.

	MK	AAM
Amorphous fraction (AF, wt %)	97 ± 1.7	83 ± 3.4
Crystalline fraction (CF, wt %)	3.0 ± 1.7	17 ± 3.4
Quartz (wt % of CF)	15.2	89.2
Muscovite (wt % of CF)	84.2	10.8
R^1^	3.09	3.34
wR^2^	4.05	5.14
R-bkg^3^	4.62	7.53
wR-bkg^4^	4.05	5.14
wRmin^5^	2.86	2.86

The total residuals after the last refinement indicate the goodness of fit in the least squares refinement, where: ^1^R is the R-factor, which is a measure of the agreement between the observed and calculated diffraction patterns during crystallographic refinement; ^2^wR is the weighted R-factor; ^3^R-bkg is the background R-factor; ^4^wR-bkg is the weighted background R-factor; and wRmin is the lowest weighted R-factor achieved during the refinement process.

After the refinements, the crystalline and amorphous mass fraction of the samples was estimated, considering that the scattering in amorphous material is the main contribution to the background in the diffractograms^44^. The total area and the area of the refined background were calculated for each diffractogram, resulting in a crystalline/amorphous mass fraction for AAM of around 17 wt%/83wt% and for MK of around 3wt%/97 wt% (Fig. 4 and Table 2). The detailed phase-specific structural and atomic compositions will be discussed below.

### Amorphous phase composition of MK and AAM

**Fig. 5:**
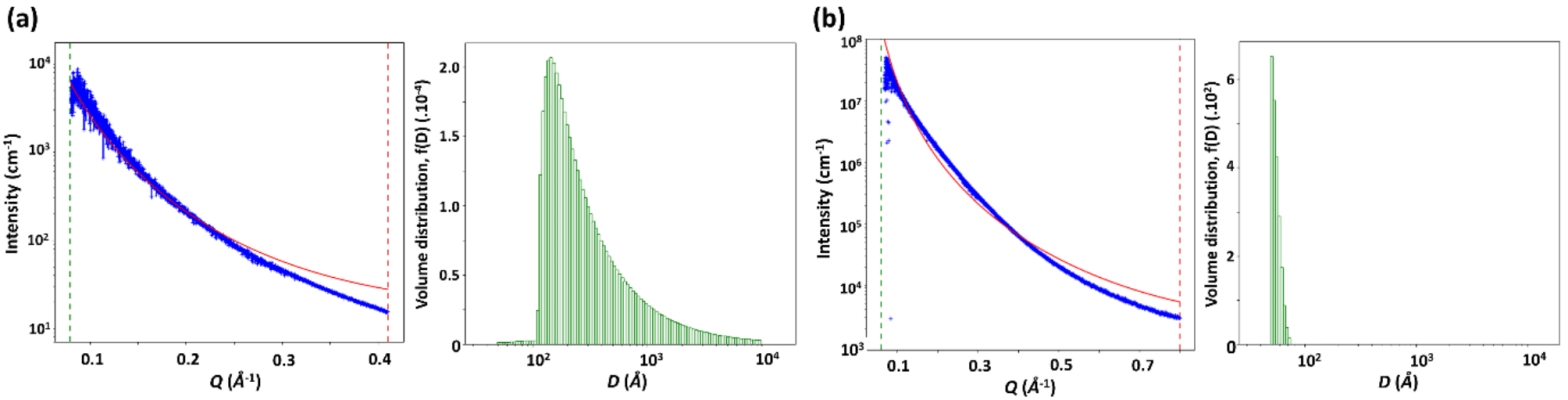
SAXS profiles and particle size distribution results from SAXS analysis of MK (**c**) and AAM (**d**).

The small angle X-ray scattering (SAXS) image data of MK and AAM were integrated using the software GSAS–II and adjusted using the Porod model (Fig. 5). The best fittings resulted in particles with the main sizes around 20 nm for MK and 5 nm for AAM, where latter presents a remarkably narrower particle size distribution than MK (Fig. 5a vs. b). Also, wide-angle x-ray scattering (WAXS) results show low-intensity Bragg reflections for AAM in contrast to MK, are presented in SI (Figure S2).

The amorphous phase in AAM is produced by chemical reaction with the alkali and is inhomogeneous and far from equilibrium, likely consisting in a mixture of amorphous NaAlSi_2_O_6_ and SiO_2_ with a ratio [Si]:[Al] 2:1. Previous molecular dynamics simulations indicate that the dissolution of MK occurs layer by layer as the Na ions penetrate the surface, and that the sublayer of Al tetrahedra is more resistant to dissolution than the sublayer of Si tetrahedra^45,46^. However, this is a debated mechanism that is dependent on composition and on the dominant cationic species (Na or K)^47,48^. Regardless, this results in heterogeneity, with multilayer structures remaining due to incomplete reaction. According to microscopy imaging, MK undergoes expansion of the layers during dissolution. Since the dissolution has already been covered by previous studies, here we will concentrate on the structure of the amorphous product of the dissolution reaction.

We use models of NaAlSi_2_O_6_ obtained by molecular dynamics simulations at constant temperature to understand the local chemical environments in the amorphous fraction of the AAM. The amorphous SiO_2_ structure has been extensively studied in previous literature^49^. The models were obtained using the REAX classical potential^50^ (more details can be found in the methods section). We have benchmarked the accuracy of the potential by calculating the lattice constant of cubic analcime at 0 K, which is 4% overestimated with respect to the experimental value^51^.

**Fig. 6:**
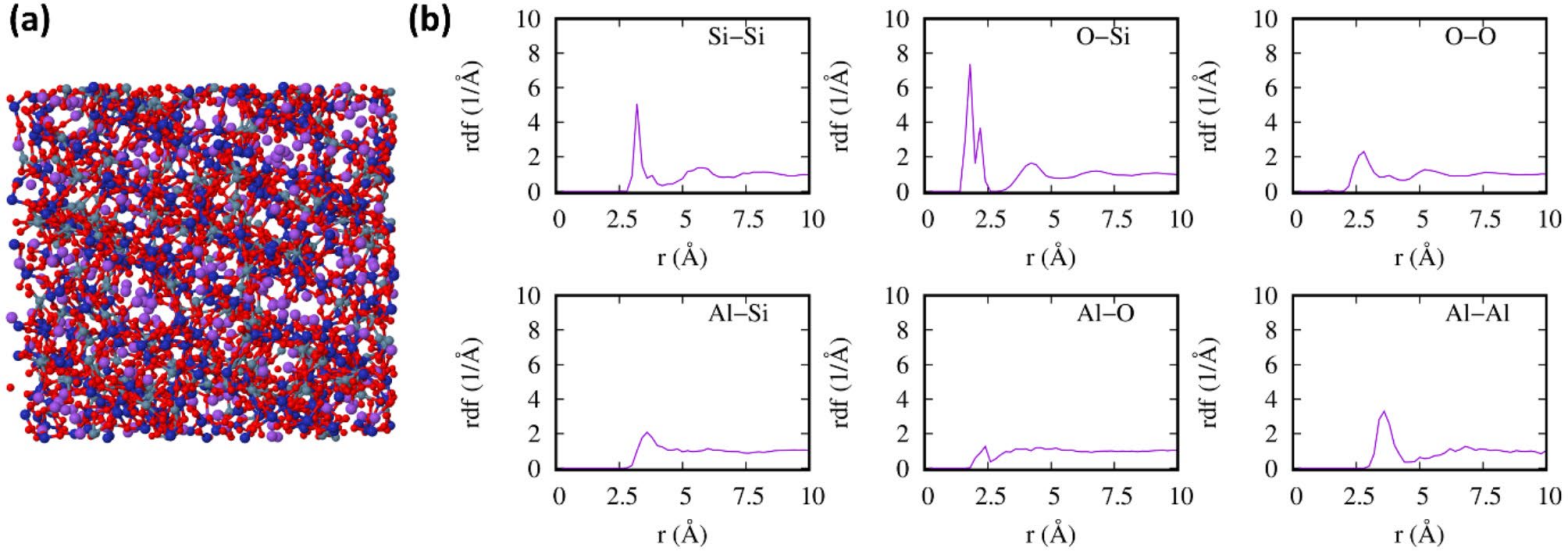
(**a**) Structure and (**b**) radial distribution functions of amorphous NaAlSi_2_O_6_. Na, Al, Si and O are represented as violet, gray, blue and red spheres, respectively.

We start by describing the amorphous NaAlSi_2_O_6_ phase, which is similar to the model previously proposed for the K-Poly(sialate-siloxo)^52^, a three-dimensional structure where both Al and Si form tetrahedra sharing the vertices. The Na atoms occupy the empty galleries or channels of the structure. A typical structure and its RDF are shown in Fig. 6a-b. The shortest bonds are Si–O at 1.79 Å, with a shoulder at 2.20 Å which is due to five-fold coordinated Si. There are additional peaks at about 4.17, 6.72 and 9.07 Å indicating that there is a correlation between the silicon tetrahedra. In contrast, in the Al-O RDF shows a peak at 2.28 Å followed by a broad feature centered at about 4.6 Å. This is due to the preference of Al tetrahedra for sharing corners with Si tetrahedra rather than with another Al tetrahedra. For the same reason, the Si–Si RDF shows multiple peaks, the first at 3.21 Å, and subsequently at about 5.7, 8.01 and 10.8 , but the Al-Al RDF shows a single sharp peak for nearest neighbors at 3.50 Å, followed by a broad feature centered at about 6.9 Å. Note that the radial distribution functions are computed for an infinite material, and therefore does not show a long-range extinction due to the particle size, which will be discussed later.

### Crystalline phase composition of MK and AAM

**Fig. 7:**
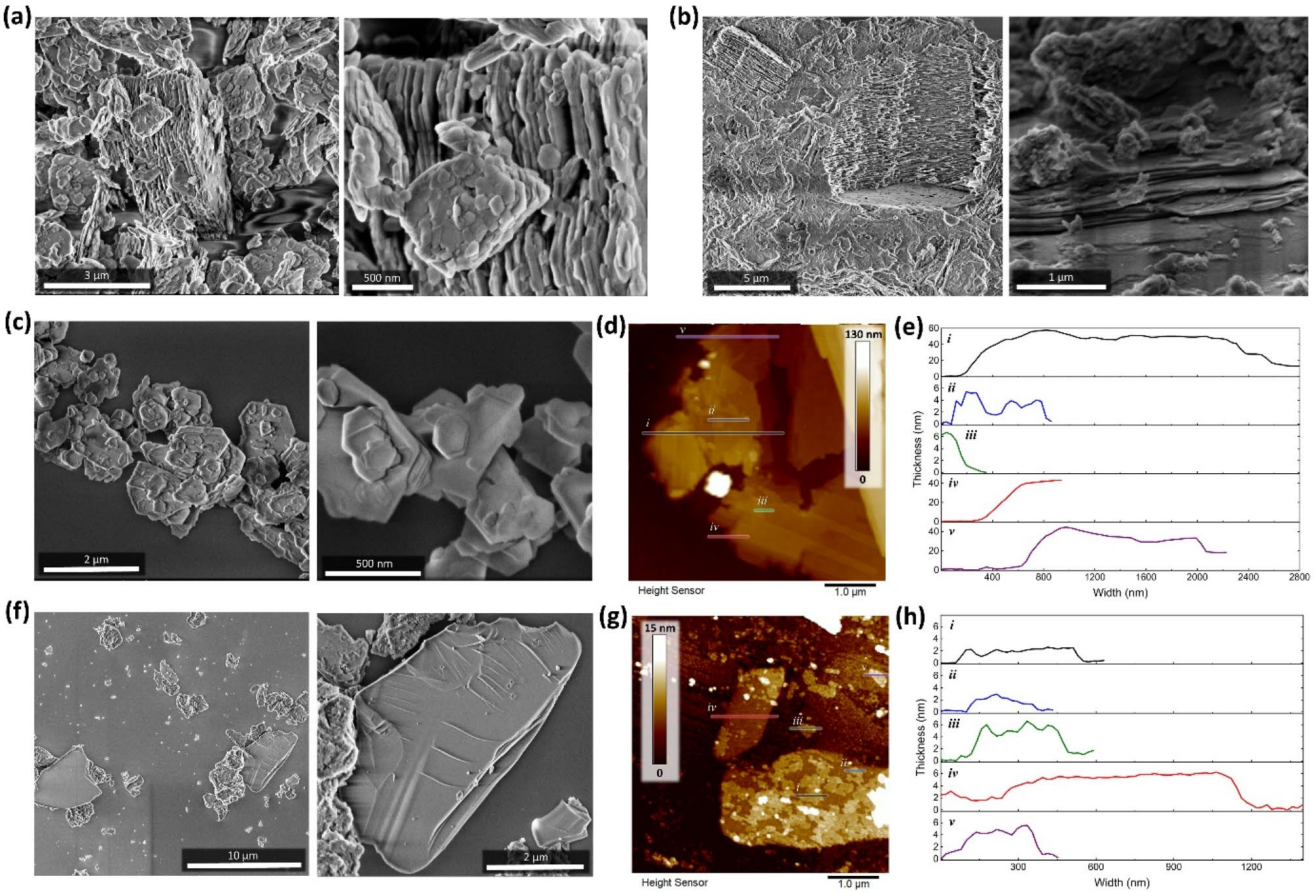
SEM images of (**a**) MK and (**b**) AAM before LBFE. (**c**,**f**) SEM and (**d**,**g**) AFM images after exfoliation, including the (**e**,**h**) thickness distribution of different isolated sheets and clusters of MK and AAM, respectively.

The crystalline phases of MK and AAM were observed using SEM, and both present very defined layered structures in their bulk conditions. While MK is constituted of more loosely and disordered stacked sheets with large size distribution (Fig. 7a), AAM presents a more compact layered structure embedded into an amorphous phase (Fig. 7b), akin to a sintered structure^53^.

For better visualization of the fundamental structure of the layered crystalline phases, MK and AAM were exfoliated using an adaptation of a liquid biphasic exfoliation system (LBFE, details in the experimental section)^54^, to decrease the in-plane crystal fracturing implied by cavitation. Moreover, LBFE allowed breaking apart the different phases of the mortar and reveal the clean remaining crystalline 2D phase of AAM by stabilizing it at the solvent’s interphase, and isolated collection. Upon exfoliation, the MK clusters become partially unstacked (Fig. 7c) revealing smaller micro-aggregates that are 40-60 nm thick, and few-layer (~ 6 nm thick) platelets with lateral sizes in the hundreds of nanometers (Fig. 7d-e). Differently, AAM aggregates break apart into wider sheet structures, followed mostly by attached amorphous debris (Fig. 7f). The clusters are an order of magnitude thinner than MK’s (Fig. 7g-h), and on top of them very fine 2D particulates can be found presenting thicknesses below 6 nm. In fact, the thinnest observed structures reach 2 nm, which is most likely a monolayer sheet.

Altogether, MK presents an overall more brittle structure and breaks apart into a random sheet size distribution, while AAM presents fewer but better preserved and larger sheets with thinner thickness profiles. In fact, AAM seems to reach close to monolayer structure while preserving (or reconstructing) a large lateral size (even after exfoliation), and its amorphous phase seems to be broken down by the process.

**Fig. 8:**
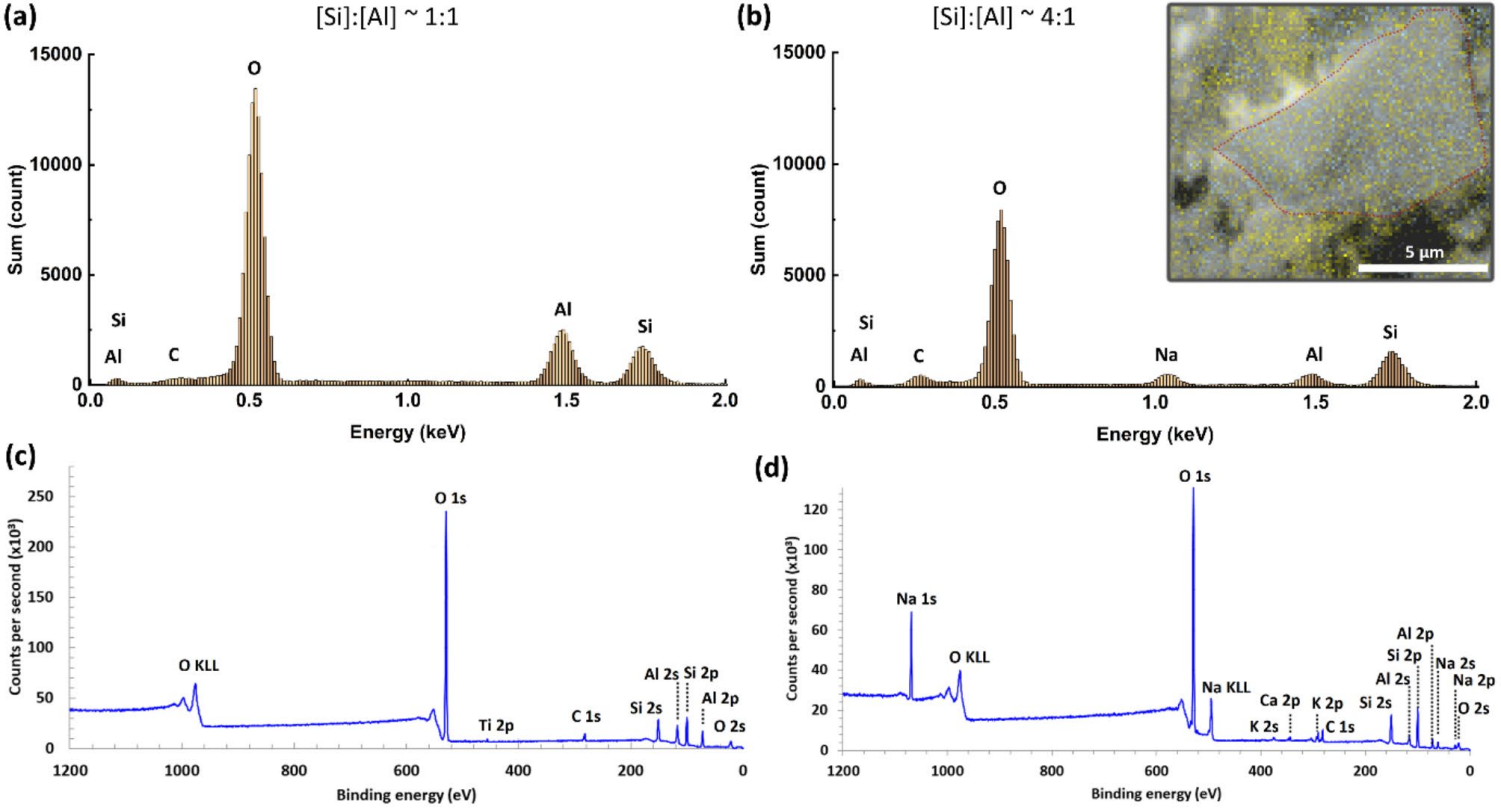
Atomic distribution of (**a**–**c**) MK and (**b**–**d**) AAM as obtained by EDX and XPS, respectively. The inset in (**b**) shows an SEM/EDX image with heterogeneous Al (blue) and Si (yellow) distribution when comparing the crystalline and amorphous phases in AAM, and the red dotted line highlights the edge limits of an AAM crystallite.

Using SEM/EDX and XPS we evaluate the atomic composition of the exfoliated/isolated crystal phases. The MK composition presents a ratio [Si]:[Al] ~ 1:1 with both EDX and XPS (Fig. 8a and c), and this ratio has only a small variation before and after exfoliation (Figure S3a). On the other hand, although AAM presents also a corroborating ratio between EDX and XPS, it has a largely increased silicon content ([Si]:[Al] ~ 4:1, Fig. 8b and d), which is due to the addition of sand, silica fume and GGBFS, and falls to half after exfoliation ([Si]:[Al] ~ 2:1, Figure S3b). The inset in Fig. 8b shows a large discrepancy in Si content when comparing the sheet area (demonstrated as the red dotted area) and the peripheric regions. This indicates that most of the Si excess is present in the amorphous phase as SiO_2_ clusters, or quartz from the SLAG and aggregates. During the alkali-activation, the dissolution of MK in the alkaline solution favors the formation of the SiO_2_ phase due to the separation of the SiO_4_ tetrahedral sheets and the AlO_6_ octahedral sheets. This could explain the excess formation of the amorphous silica found in the AAM samples. However, the crystal composition seems to also favor the inclusion of Si in the final composition after alkali activation, as [Si]:[Al] from the crystalline phase of AAM is twice as large as MK’s (see SI, Figure S3).

The 2D order of the residual nanocrystals affects the formation of the amorphous surrounding of the crystalline phases in AAM, as observed by the SAXS profiles and corresponding particle size distributions. The SAXS intensity follows a power-law decay, in which the high-q tail of scattering is caused by particles with a narrow distribution of sizes between ~ 30 and ~ 50 nm (Fig. 5b), which is consistent with the reported formation of SiO_4_ and AlO_4_ tetrahedra in the structure of AAMs^43^. Furthermore, these sizes correspond to a solidification after a reaction in a liquid solution, which could allow for the nucleated/templated growth of some of this material as a continuous crystalline phase or the direct crosslinking between crystalline and amorphous phases. Such crystal growth behavior during alkali-activation were previously observed^55^. However, in our systems they seem to be nucleated by the residual crystals, forming a Si-rich and heterogeneous composition in the transition between the crystal and amorphous phases (see Fig. 8b, inset). However, we cannot exclude the possibility that some of the crystals are residual, from the mortar powder fillers.

## Conclusion

We have shown that a crystalline 2D material, namely kaolinite, can be converted into a 3D amorphous material with short range 2D order by chemical reaction in an alkaline medium. This dimensional transformation, combined with an order to disorder mechanism, is possible by the consumption of the 2D material due to its large surface chemical reactivity. However, the chemical reaction is self-limiting resulting in a complex structure, an intermediate between a 3D glass and a 2D crystal, where the nanoscale 2D crystalline grains are surrounded by an amorphous 3D structure with short range order. The final material can have many applications including as a green cement since it does not produce carbon dioxide during its transformation, as is the case of traditional Portland cement.

## Materials and methods

### Materials

The precursor and reagent used for the synthesis of AAM are a commercially available metakaolin under the brand name Metamax®. (ii) The liquid alkali silicate is a mixture of Na and K silicates, M = 1.5 and water content of 56 wt%. Na and K silicates were supplied by ISPL (www.ispl.com.sg, brand ISPL 51) and Noble Alchem (http://noblealchem.com, brand PSLP K13), respectively. (iii) The silica fume was supplied by Elkem (www.elkem.com, brand 920D). (iv) The Slag (GGBFS) was supplied by Engro (www.engro-global.com, brand P8000). (v) NaOH and KOH were supplied by Sigma Aldrich.

### Methods

#### Formulation and processing of the reagent

The reagent used for binder synthesis is a mixture of Na and K liquid silicates (composition described in the materials section), the modulus M of both being ~ 1.5. The original M of the as-delivered Na and K silicates have been altered to M = 1.5 by adding NaOH and KOH, respectively. After correction and mixing, the resulting alkali solution was left to rest for at least 24 h prior use.

#### Formulation and processing of AAM (binder)

The AAM used as a binder comprises metakaolin, composite liquid alkali silicate (described above), and silica fume and GGBFS as minor components. Materials in the mix design were proportioned to result in a theoretical composition in which (molar ratios) SiO_2_/Al_2_O_3_ = 4, Al_2_O_3_/(Na,K)_2_O = 1, CaO/SiO_2_ = 0.05, and H_2_O/(Na,K) = 11. More specifically for the composition characterized in this study, the mass fractions used were as follows: Metakaolin: 33.5 wt%, liquid alkali silicates: 52 wt% (M = 1.5), silica fume: 7 wt%, GGBFS: 7.5 wt%. After mixing the solids and the liquid silicate using an IKA EUROSTAR 40 mechanical stirrer at 600 rpm, the stirring was kept long enough to remove all visible agglomerates.

#### Formulation and processing of the mortar

The mortar, which is a combination of a cementitious agent and aggregates, was prepared mixing the binder above described and river sand (quartz, d_90%_ = 2 mm, d_50%_ = 0.5 mm and d_10%_ = 0.04 mm) in the proportion of 1L of binder to 3L of aggregates, using a Matest E092N lab mortar mixer (Matest, Italy). After mixing, the mortar was cast in prismatic molds (15 × 15x30mm) and left to cure at room temperature for 28 days before use. All characterization of the powder AAM were performed using this mortar after finely grinded.

#### Liquid biphasic exfoliation (LBFE)

MK and AAM were exfoliated using an adaptation of a liquid biphasic exfoliation system ^4,54^, containing water and dichloromethane (DCM) as the exfoliation media. Briefly, 1 g of MK or finely grinded mortar containing AAM were added to a 250 mL round bottom flask containing 90 mL of water and 10 mL of DCM. The round bottom flask with the mixture was then sonicated, using a 32 kHz/500W bath ultrasound (Bandelin) for 4 h, with a constant temperature of 10 °C. After exfoliation, the dispersion was left to rest overnight, for the large particulates to settle, forming a phase separation at the bottom. Then, 100μL of the water-phase (top) supernatant was collected close from the solvent interphase and dripped on SiO_2_ coated Si substrates, which were left to dry at 45 °C for ~ 6 h. These substrates were used for AFM and SEM characterizations.

### Characterization methods

*XRD:* X-ray powder diffraction data were collected in a standard laboratory diffractometer Rigaku-Dairix with CuKα radiation and Bragg–Brentano geometry. The diffractometer was set in step-scan mode with steps of 0.01° and with a counting time of 5 s. The powder samples were prepared by back-mounting to avoid preferential orientation. Solid samples were cut into slices with the proper dimensions to collect diffraction data in the same conditions. The QPA and crystal structure refinement were performed by the Rietveld method using the General Structure Analysis System-II (GSAS – II). The software uses an axial-divergence-broadened pseudo-Voigt as the peak profile function and allows refinement of the parameters of Gaussian and Lorentzian widths for each individually fitted peak. The instrumental parameters for profile refinement were initially determined using the diffraction data of a silicon standard sample. This allows the refinement of physically meaningful parameters for the analyzed samples^56^, producing reliable quantitative measurements without the use of internal standards^57^.

*SAXS/WAXS:* Powder samples were analyzed for Wide and Small-angle X-ray scattering (WAXS and SAXS) using a standard laboratory small- and wide-angle X-ray scattering equipment Xenocs Xeuss 2.0 SAXS/WAXS with wavelength λ = 1.5418 Å. The samples were disposed of in stainless steel washers of 1 cm diameter and covered with a Kapton tape, which is commonly used for its high transparency to X-rays in the SAXS region. The analysis’s q-range extends from 0·05 Å^−1^ to 0.9 Å^−1^.

*SEM-FESEM:* The characterization by field emission scanning electron microscopy (FESEM) with energy dispersive x-ray (EDX) was performed at an equipment FEI Verios 460 L. Samples of the precursor MK and AAM were coated with a gold layer of approximately 5 nm thickness and analyzed using Carl Zeiss AG—SUPRA 40 equipment.

*Elemental analysis* to quantify carbon, nitrogen, hydrogen, sulphur, and oxygen (CNHS-O) was performed using an organic elemental analyzer Vario El cube (Elementar – Germany).

*AFM:* Drop-casted dispersions on SiO2/Si substrate were imaged using a Bruker Dimension FastScan® Atomic Force Microscope (AFM) in a tapping mode with a silicon tip on silicon nitride cantilever (T: 0.6 um, L: 27 µm, W: 32 µm, f0: 1400 kHz, k: 18 N/m). The images were obtained with a pixel resolution of 512 sample/line. The image processing and height profiles were performed using NanoScope Analysis software.

*X-ray photoelectron spectroscopy (XPS):* The powder samples were analyzed by XPS on a Thermo Escalab 250Xi using AlKα radiation. Survey spectra were collected with a pass energy of 150 eV and a step size of 1 eV. High-resolution spectra were collected with a pass energy of 30 eV and a step size of 0.05 eV. During each scan, an ion gun was utilized to neutralize the charging effects.

*Chemical elemental analysis (CNHS-O and ICP-OES):* Quantification of carbon, nitrogen, hydrogen, sulphur, and oxygen (CNHS-O) was performed in an organic elemental analyzer, Vario El cube -Elementar. For analysis by Inductively Coupled Plasma-Optical Emission Spectrometer (ICP-OES), the powder samples were digested with HNO_3_/HCl (3:1) in a microwave oven at 240 °C for 15 min and topped up to 14 mL with H_2_O. A Perkin Elmer Avio 500 equipment was used for the analysis of the metal species. In total, 65 atomic species were analyzed, while 28 were detectable and the rest were below the detection limits of the method.

### Computational methods

#### Density functional theory calculations

We have modelled kaolinite and MK using density functional theory calculations based on the models proposed in Ref.^34^. The calculations were carried out using the SIESTA package^58^. The core electrons were modelled using pseudopotentials of the Troullier-Martins type^59^. The bases set for the Kohn–Sham states are linear combinations of numerical atomic orbitals (a single zeta polarized basis for hydrogen and double zeta polarized basis for all other species). The charge density was projected on a real-space grid with an equivalent cut-off energy of 250 Ry to calculate the exchange–correlation and Hartree potentials. Structural relaxations were performed using conjugate gradient optimization.

The lattice parameters of kaolinite, obtained with the PBE functional^60^, are more accurate than those obtained with the LMKLL functional, which is more commonly used for van der Waals bonded systems^61^ (see Table 1). Both functionals predicted accurately the inter-layer distance. We therefore will use the PBE functional for the calculations unless otherwise stated.

#### Classical molecular dynamics simulations

Classical molecular dynamics simulations were performed using the LAMMPS code^62^, using the REAX potential developed by^50^. The amorphous structure of NaAlSi_2_O_6_ was prepared by starting from a 3 × 3x3 supercell of analcime with 6 × 3^2^ formula units, which was first expanded to double its equilibrium size for an initial amorphization step, followed by annealing at 900 K for 8 ns at the fixed volume corresponding to the low temperature lattice constant, following by a 20 ps quench to 300 K. The amorphous structure of NaAlSi_3_O_8_ was prepared by starting from a 3 × 2x3 supercell of albite. It was initially amorphized by a short annealing with all the lattice parameters expanded by double. Then, it was annealed at 900 K at the experimental volume, with orthogonal lattice parameters 24.4, 26.0 and 19.2 Å.

## Electronic supplementary material

Below is the link to the electronic supplementary material.

Supplementary Material 1

Supplementary Material 2

## Data Availability

The datasets used and/or analyzed during the current study are available from the corresponding author upon reasonable request.
