# Supplementary material for: From 2D kaolinite to 3D amorphous cement
Source: Sci Rep. 2025 Jan 11;15:1669. doi: 10.1038/s41598-024-81882-1 (PMC11724968; doi:10.1038/s41598-024-81882-1)
Supplement: Supplementary file 2 — Supplementary Material 2 [file 41598_2024_81882_MOESM2_ESM.pdf]

From 2D kaolinite to 3D amorphous cement

Juan A. G. Carrio[1], Ricardo K. Donato[1], Alexandra Carvalho[1,2], Gavin K. W. Koon[1], Katarzyna Z. Donato[1], Xin Hui Yau[1], Dmytro Kosiachevskyi[1], Karen Lim[1], Vedarethinam Ravi[1], Josny Joy[1], Kelda Goh[1], Jose Vitorio Emiliano[1], Jerome E. Lombardi [1], A. H. Castro Neto[1,2,3*]

[1] Centre for Advanced 2D Materials, National University of Singapore, 117546, Singapore.
[2] Institute for Functional Intelligent Materials (I-FIM), National University of Singapore, 117544, Singapore.
[3] Department of Materials Science and Engineering, National University of Singapore, 117575, Singapore.

*Corresponding author: c2dhead@nus.edu.sg

Keywords: clays, 2D materials, metakaolin, alkali-activation, cement

Supplementary Information

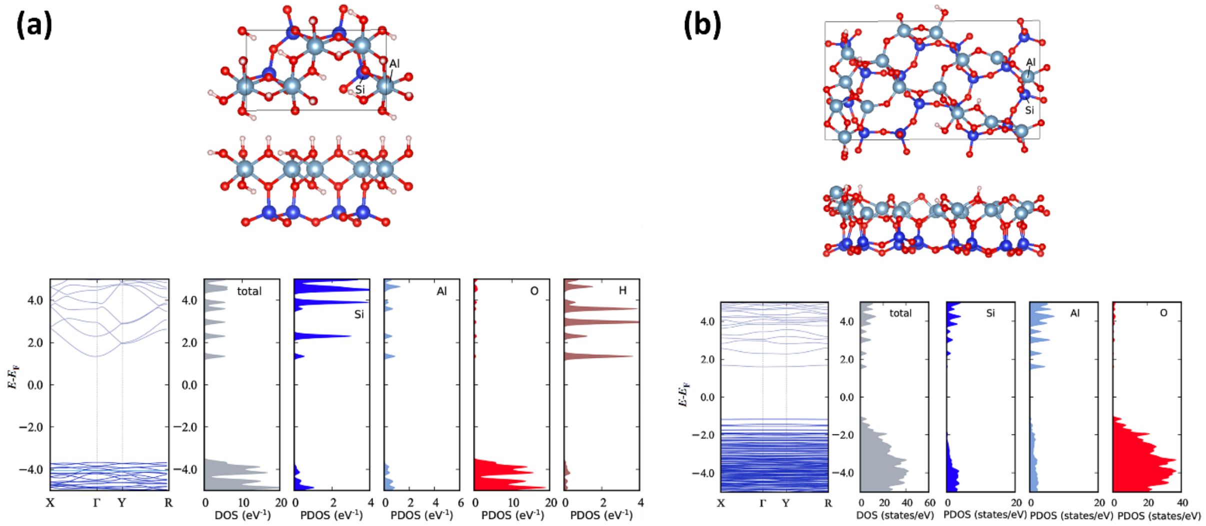

Figure S1. (a) Kaolinite and (b) MK monolayer bandstructures

Table S1: Bulk atomic composition by ICP/IOS analysis of AAM and MK.

Detectable atoms in order of abundance (in mass ppm)

Atom	*AAM*	*MK*	*Atom*	*AAM*	*MK*
Si	160388	86091	Cu	49	12
Al	27790	224745	Ba	41	91
Na	20821	2033	Zr	40	73
K	9931	938	V	27	144
Ca	4583	106	Zn	20	19
Fe	1967	2157	Ce	19	114
Ti	1509	6767	Sr	17	41
Mg	995	152	Li	13	30
S	405	173	Mo	5	ND
Cr	142	100	Y	5	6
Ga	135	617	Co	3	16
Mn	94	3	Sc	1	14
B	71	40	P	ND	241
Ni	56	24	Be	ND	3

Atoms analysed but below detection level both in AAM and MK. As, Bi, In, Se, Cd, Pb, Hg, Ag, Tl, Sb, Ge, Hf, Nb, Re, Ta, Sn, W, Dy, Er, Gd, Ho, La, Lu, Nd, Pr, Sm, Tb, Th, Tm, U, Yb, Au, Ir, Pd, Pt, Rh and Ru.

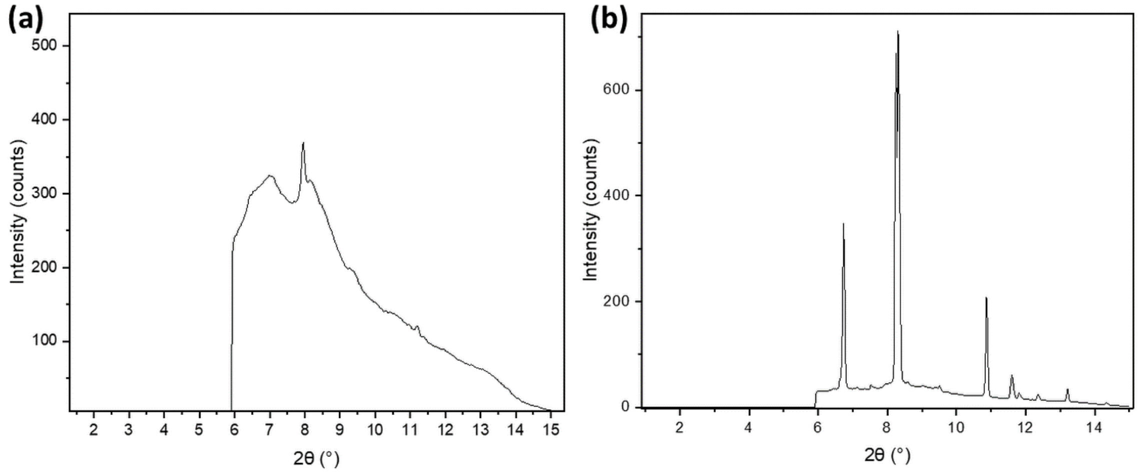

Figure S2. Amorphous phase structure as observed by the WAXS profiles for (a) MK and (b) AAM.

(a) MK

BEFORE

Metakaolin	Atomic% Si	Atomic% Al
1	23.54	24.93
2	18.21	20.35
3	17.05	19.30
4	17.05	19.41
5	17.98	20.55
**Average**	**18.766**	**20.908**

Si : Al = 1 : 1.11

AFTER

Metakaolin	Atomic% Si	Atomic% Al
1	18.04	16.09
2	22.44	14.36
3	19.27	15.81
4	16.74	16.54
5	16.46	16.42
**Average**	**18.59**	**15.844**

Si : Al = 1.17 : 1

(b) AAM

BEFORE

Geo-polymer	Atomic% Si	Atomic% Al
1	23.61	6.43
2	26.13	4.37
3	19.50	7.83
4	23.14	7.63
5	27.29	4.00
**Average**	**23.934**	**6.052**

Si : Al = 3.95 : 1

AFTER

Geo-polymer	Atomic% Si	Atomic% Al
1	25.65	6.73
2	19.37	9.52
3	21.47	10.88
4	16.89	8.89
5	17.61	9.69
**Average**	**20.198**	**9.142**

Si : Al = 2.21 : 1

Figure S3. Elemental (Si and Al) composition of (a) MK and (b) AAM crystalline phases obtained by EDX, before and after removal of most of the amorphous phase using LBFE (see experimental section). The individual values are atomic abundances obtained in different points in different crystalline phases of the same image.

Table S2: Refined parameters and space group of phases Muscovite and Quartz (see also CIF file).

Parameter	Muscovite	Quartz
**space group**	C 2/c	P 31 2 1
**a**	8.960(5)	4.9218(2)
**b**	5.046(2)	4.92176
**c**	19.94(1)	5.4101(2)
**α**	90	90
**β**	96.68(4)	90
**γ**	90	120
**cell volume**	895.4(5)	113.494(3)
